# Supplementary material for: Biodegradation of Microtoxic Phenylpropanoids (Phenylpropanoic Acid and Ibuprofen) by Bacteria and the Relevance for Their Removal from Wastewater Treatment Plants
Source: Genes (Basel). 2023 Feb 9;14(2):442. doi: 10.3390/genes14020442 (PMC9956071; doi:10.3390/genes14020442)
Supplement: Supplementary file 1 [file genes-14-00442-s001.zip › genes-2195262-supplementary.pdf]

Supplementary Materials

Table S1. Primer list

Name	Sequence (5′-3′)	Target gene	pRW412a region*
REV	CAGGAAACAGCTATGAC	Sequencing primer	-
UNIV	GTTTTCCCAGTCACGAC	Sequencing primer	-
GM3F	AGAGTTTGATCATGGC	16S rRNA gene	-
GM4R	TACCTTGTTACGACTT	16S rRNA gene	-
IpfAfor	GTCTTCGTTGGTCACGACTC	*ipfA*	III
IpfBrev	TTCACTCGCGTGACCATG	*ipfB*	III
IpfDfor	ACCATGGTCACGCGAGTGA	*ipfD*	III
IpfDrev	GGTGCTATCCATCAATGGCTCC	*ipfD*	III
IpfFfor	GTTATGTTGGCAAGAGACCTGG	*ipfF*	III
IpfFrev4	CTTTCTTGCGTCAGGATGGC	*ipfF*	III
HMSDFor-	TCCGGCATTTCATCGATG	*ipfM*	I
HMSDRev	ATGATGGCGCTGTTCTTGC	*ipfM*	I
IpfIfor-	GAGCGTTTGAAGTCTTGGGC	*ipfI*	II
IpfIrev	CAATCACGGTGCGTGTGAAG	*ipfI*	II
DDFor	GACGGCGGAAGTTTTGAA	Dienoate dehydrogenase gene (DD)	IV
DDRev	CTTGACGACGACGGTGTT	Dienoate dehydrogenase gene (DD)	IV
DsbC2For-	CACAATGCATCCGCTACG	Thiol:disulfide gene involved in conjugative transfer (DsbC)	V+I
DsbC2Rev	CGGATCCTTCGCACAATAG	Thiol:disulfide gene involved in conjugative transfer (DsbC)	V+I

* Roman numerals denote regions delimitated by IS*6* elements as indicated for pIBU218 of *R. wittichii* MPO218 (Aulestia et al., 2021).

Table S2. PCR amplification

Target gene	pRW412a region*	*R. wittichii* RW1	*S. granuli* RW412	*R. wittichii* RW421	*P.citronellolis* RW422	*P.citronellolis* RW423	*P.citronellolis* RW424
ipfAB	III	ND	+	+	+	+	-
ipfD	III	ND	+	+	+	+	-
ipfF	III	ND	+	+	+	+	-
ipfM	I	-	+	-	+	+	-
ipfI	II	-	+	+	+	+	-
DD	IV	-	+	+	+	+	-
DsbC	V+I	-	+	-	+	+	-

* Roman numerals in analogy to that used for pIBU218 of *R. wittichii* MPO218 (Aulestia et al., 2021).

ND: not determined

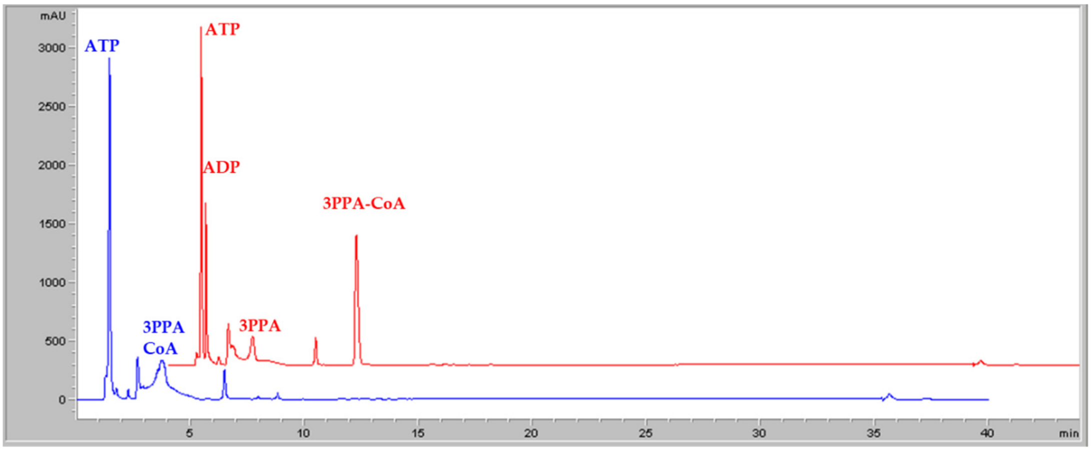

Figure S1. Overlay of HPLC chromatograms at 210 nm of the reaction components of 3-phenylpropanoic acid (3PPA) with IpfF initially (in blue) and after 35 minutes (in red).

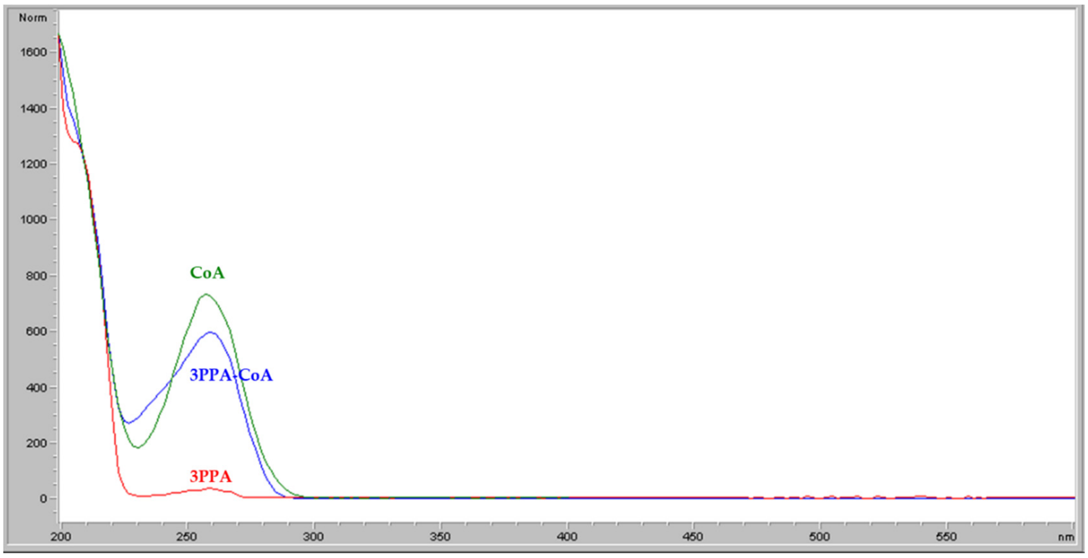

Figure S2. Overlay of spectra (200-600 nm) of the 3-phenylpropanoyl-CoA product (3PPA-CoA, in blue) compared to those of 3-phenylpropanoic acid (3PPA, in red) and coenzyme A (CoA, in green).

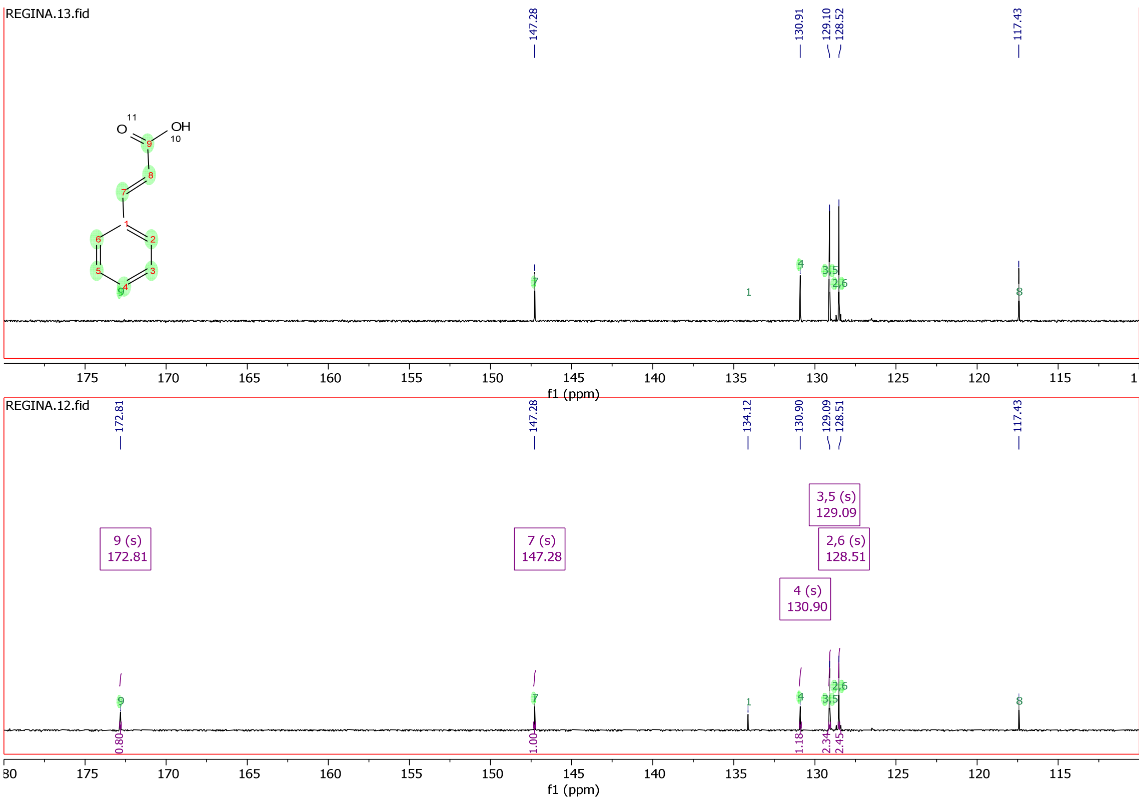

Figure S3. ^{13}C NMR (BB and DEPT135) spectrum of cinnamic acid, recorded at 151 MHz in deuterochloroform.

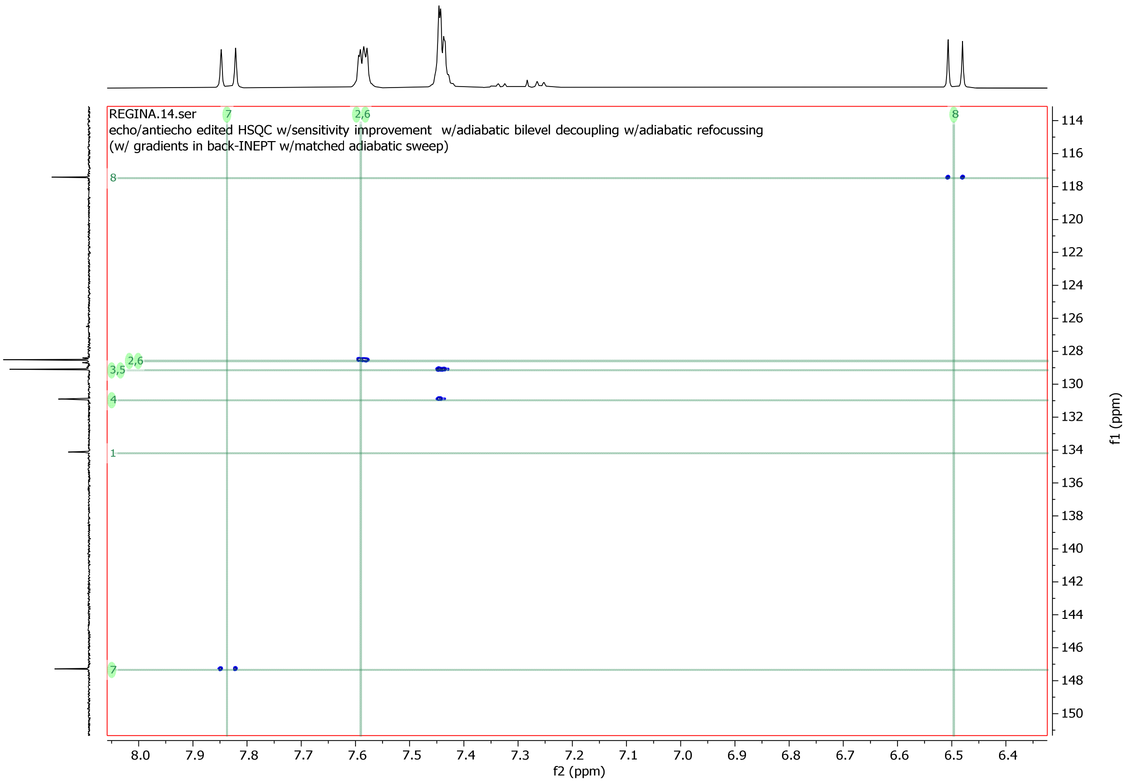

Figure S4. ^{1}H-^{13}C HSQC 2D-NMR spectra of cinnamic acid.

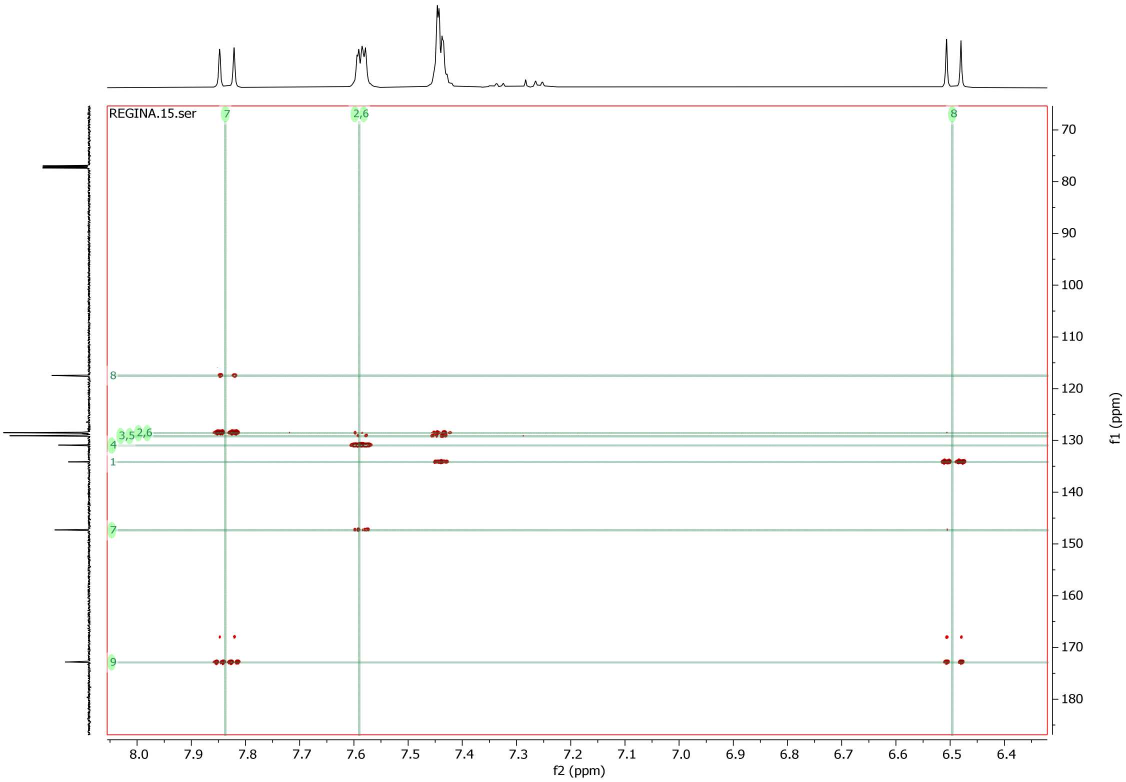

Figure S5. ^{1}H-^{13}C HMBC 2D-NMR spectra of cinnamic acid to assign the quaternary carbons.
